# Automated Taxonomic Identification of Insects with Expert-Level Accuracy Using Effective Feature Transfer from Convolutional Networks

**DOI:** 10.1093/sysbio/syz014

**Published:** 2019-03-02

**Authors:** Miroslav Valan, Karoly Makonyi, Atsuto Maki, Dominik Vondráček, Fredrik Ronquist

**Affiliations:** 1 Savantic AB, Rosenlundsgatan 52, 118 63 Stockholm, Sweden; 2 Department of Bioinformatics and Genetics, Swedish Museum of Natural History, Frescativagen 40, 114 18 Stockholm, Sweden; 3 Department of Zoology, Stockholm University, Universitetsvagen 10, 114 18 Stockholm, Sweden; 4 Disciplinary Domain of Science and Technology, Physics, Department of Physics and Astronomy, Nuclear Physics, Uppsala University, 751 20 Uppsala, Sweden; 5 School of Electrical Engineering and Computer Science, KTH Royal Institute of Technology, Stockholm, SE-10044 Sweden; 6 Department of Zoology, Faculty of Science, Charles University in Prague, Viničná 7, CZ-128 43 Praha 2, Czech Republic; 7 Department of Entomology, National Museum, Cirkusová 1740, CZ-193 00 Praha 9 - Horní Počernice, Czech Republic

## Abstract

Rapid and reliable identification of insects is important in many contexts, from the detection of disease vectors and invasive species to the sorting of material from biodiversity inventories. Because of the shortage of adequate expertise, there has long been an interest in developing automated systems for this task. Previous attempts have been based on laborious and complex handcrafted extraction of image features, but in recent years it has been shown that sophisticated convolutional neural networks (CNNs) can learn to extract relevant features automatically, without human intervention. Unfortunately, reaching expert-level accuracy in CNN identifications requires substantial computational power and huge training data sets, which are often not available for taxonomic tasks. This can be addressed using feature transfer: a CNN that has been pretrained on a generic image classification task is exposed to the taxonomic images of interest, and information about its perception of those images is used in training a simpler, dedicated identification system. Here, we develop an effective method of CNN feature transfer, which achieves expert-level accuracy in taxonomic identification of insects with training sets of 100 images or less per category, depending on the nature of data set. Specifically, we extract rich representations of intermediate to high-level image features from the CNN architecture VGG16 pretrained on the ImageNet data set. This information is submitted to a linear support vector machine classifier, which is trained on the target problem. We tested the performance of our approach on two types of challenging taxonomic tasks: 1) identifying insects to higher groups when they are likely to belong to subgroups that have not been seen previously and 2) identifying visually similar species that are difficult to separate even for experts. For the first task, our approach reached $CDATA[$CDATA[$>$$92% accuracy on one data set (884 face images of 11 families of Diptera, all specimens representing unique species), and $CDATA[$CDATA[$>$$96% accuracy on another (2936 dorsal habitus images of 14 families of Coleoptera, over 90% of specimens belonging to unique species). For the second task, our approach outperformed a leading taxonomic expert on one data set (339 images of three species of the Coleoptera genus *Oxythyrea*; 97% accuracy), and both humans and traditional automated identification systems on another data set (3845 images of nine species of Plecoptera larvae; 98.6 % accuracy). Reanalyzing several biological image identification tasks studied in the recent literature, we show that our approach is broadly applicable and provides significant improvements over previous methods, whether based on dedicated CNNs, CNN feature transfer, or more traditional techniques. Thus, our method, which is easy to apply, can be highly successful in developing automated taxonomic identification systems even when training data sets are small and computational budgets limited. We conclude by briefly discussing some promising CNN-based research directions in morphological systematics opened up by the success of these techniques in providing accurate diagnostic tools.

Rapid and reliable identification of insects, either to species or to higher taxonomic groups, is important in many contexts. Insects form a large portion of the biological diversity of our planet, and progress in the understanding of the composition and functioning of the planet’s ecosystems is partly dependent on our ability to effectively find and identify the insects that inhabit them. There is also a need for easy and accurate identification of insects in addressing concerns related to human food and health. Such applications include the detection of insects that are pests of crops (FAO 2015), disease vectors (WTO 2014), or invasive species (GISD 2017).

Identifying insects is hard because of their immense species diversity [more than 1.02 million species described to date (Zhang 2011)] and the significant variation within species due to sex, color morph, life stage, etc. With some training, one can learn how to distinguish higher taxonomic groups, such as orders, but already at the family level the task becomes quite challenging, even for experts, unless we restrict the problem to a particular life stage, geographic region, or insect order. Generally speaking, the lower the taxonomic level, the more challenging the identification task becomes (Fig. 1). At the species level, reliable identification may require years of training and specialization on one particular insect taxon. Such expert taxonomists are often in short demand, especially for groups that are not showy and attractive, and their time could be better spent than on routine identifications.

**Figure 1.**
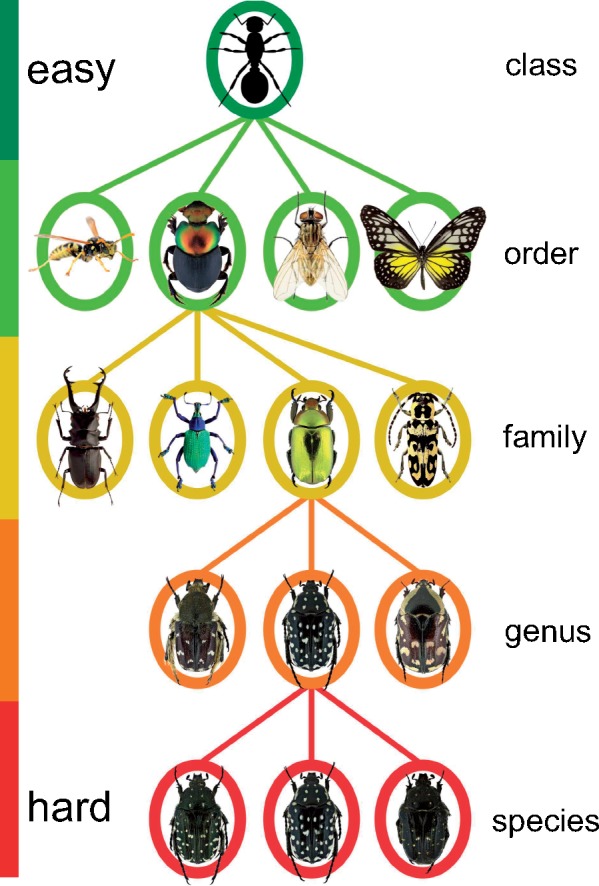
A schematic illustration of the taxonomy of insects. The full tree is organized into hierarchical ranks; it contains approximately 1.02 million known species and several millions that remain to be described. Classifying a specimen to a group of higher rank, such as order, is usually relatively easy with a modest amount of training. The challenge and amount of required expertise increases considerably (transition from green to red) as the taxonomic rank is lowered.

For these reasons, there has long been an interest in developing automated image-based systems for insect identification (Schröder et al. 1995; Weeks et al. 1997, 1999a,1999b; Gauld et al. 2000; Arbuckle et al. 2001; Watson et al. 2003; Tofilski 2004, 2007; ONeill 2007; Steinhage et al. 2007, Francoy et al. 2008; Yang et al. 2015; Feng et al. 2016; Martineau et al. 2017). Common to all such systems designed to date is that they depend on handcrafted feature extraction. “Handcrafted” or “hand-engineered” are standard terms in machine learning and computer vision referring to the application of some process, like an algorithm or a manual procedure, to extract relevant features for identification from the raw data (images in our case). Examples of features that have been used for taxonomic identification include the wing venation pattern, the relative position of wing vein junctions, and the outline of the wing or of the whole body. Although many of these systems achieve good identification performance, the need for special feature extraction tailored to each task has limited their use in practice.

In recent years, deep learning (DL) and convolutional neural networks (CNNs) have emerged as the most effective approaches to a range of problems in automated classification (LeCun et al. 2015; Schmidhuber 2015), and computer vision is one of the fields where these techniques have had a transformative impact. The basic ideas have been around for a long time (Fukushima 1979, 1980; Fukushima et al. 1983) but a significant increase in the complexity and size of the neural networks and a huge increase in the volume of data used for training have generated spectacular advances in recent years. These developments, in turn, would not have been possible without the extra computational power brought by modern graphical processing units (GPUs).

In contrast to traditional approaches of machine learning, requiring handcrafted feature extraction, DL and CNNs enable end-to-end learning from a set of training data. In end-to-end learning, the input consists of labeled raw data, such as images, nothing else. The images may even represent different views, body parts, or life stages—the CNN automatically finds the relevant set of features for the task at hand. CNNs have been particularly successful in image classification tasks, where large labeled training sets are available for supervised learning. The first super-human performance of GPU-powered CNNs (Cireşan et al. 2011) was reported in 2011 in a traffic sign competition (Stallkamp et al. 2011). The breakthrough came in 2012, when a CNN architecture called AlexNet (Krizhevsky et al. 2012) outcompeted all other systems in the ImageNet Large Scale Visual Recognition Challenge (Russakovsky et al. 2015), at the time involving 1.3 million images divided into 1000 categories, such as “lion,” “cup,” “car wheel,” and different breeds of cats and dogs. Since then, CNN performance has improved significantly thanks to the development of deeper, more complex neural network architectures, and the use of larger data sets for training. Open-source licensing of DL development frameworks has triggered further methodological advances by attracting a vast developer community.

Training a complex CNN from scratch to performance levels that are on par with humans requires a huge set of labeled images and consumes a significant amount of computational resources, which means that it is not realistic currently to train a dedicated CNN for most image classification tasks. However, in the last few years, it has been discovered that one can take advantage of a CNN that has been trained on a generic image classification task in solving a more specialized problem using a technique called *transfer learning* (Caruana 1995; Bengio 2011; Yosinski et al. 2014; Azizpour et al. 2016). This reduces the computational burden and also makes it possible to benefit from the power of a sophisticated CNN even when the training set for the task at hand is moderate to small.

Two variants of transfer learning have been tried. In the first, *fine-tuning*, the pretrained CNN is slightly modified by fine-tuning model parameters such that the CNN can solve the specialized task. Fine-tuning tends to work well when the specialized task is similar to the original task (Yosinski et al. 2014), but it may require a fair amount of training data and computational power. It is also susceptible to overfitting on the specialized task when the data sets are small because it may incorrectly associate a rare category with an irrelevant feature, such as a special type of background, which just happens to be present in the few images of that category in the training set.

The second variant of transfer learning is known as *feature transfer*, and involves the use of the pretrained CNN as an automated feature extractor (Donahue et al. 2014; Oquab et al. 2014; Razavian et al. 2014; Zeiler and Fergus 2014; Azizpour et al. 2016; Zheng et al. 2016). The pretrained CNN is exposed to the training set for the specialized task, and information is then extracted from the intermediate layers of the CNN, capturing low- to high-level image features; see description of the CNN layer architecture below). The feature information is then used to train a simpler machine learning system, such as a support vector machine (SVM) (Cortes and Vapnik 1995), on the more specialized task. Feature transfer in combination with SVMs tends to work better than fine-tuning when the specialized task is different from the original task. It is computationally more efficient, works for smaller image sets, and SVMs are less susceptible to overfitting when working with imbalanced data sets, that is, data sets where some categories are represented by very few examples (He and Garcia 2009).

Sophisticated CNNs and transfer learning have been used successfully in recent years to improve the classification of some biological image data sets, such as “Caltech-UCSD Birds-200-2011” (Birds-200-2011) (Wah et al. 2011) (200 species, 40–60 images per species) and “102 Category Flower Data set” (Flowers-102) (Nilsback and Zisserman 2008) (102 flower species commonly occurring in the UK, 40–258 images per species) (Table 1). Similar but larger data sets contributed by citizen scientists are explored in several ongoing projects, such as Merlin Bird ID (Van Horn et al. 2015), Pl@ntNet (Joly et al. 2014) and iNaturalist (web application available at http://www.inaturalist.org). These data sets involve outdoor images of species that are usually easy to separate for humans, at least with some training, and the automated identification systems do not quite compete in accuracy with human experts yet.

**Table 1.** Comparison of the performance of some automated image identification systems prior to CNNs and some recent state-of-the-art CNN-based methods on two popular fine-grained data sets (i.e., data sets with categories that are similar to each other), Bird-200-2011 (Wah et al. 2011), and Flower-102 (Nilsback and Zisserman 2008)

Methods	Bird	Flower	References
Pre-CNN methods			
Color+SIFT	26.7	81.3	(Khan et al., 2013)
GMaxPooling	33.3	84.6	(Murray and Perronnin, 2014)
CNN-based techniques			
CNNaug-SVM	61.8	86.8	(Razavian et al., 2014)
MsML	67.9	89.5	(Qian et al., 2014)
Fusion CNN	76.4	95.6	(Zheng et al., 2016)
Bilinear CNN	84.1	—	(Lin et al., 2015)
Refined CNN	86.4	—	(Zhang et al., 2017)

*Note*: All CNN-based methods used pretrained VGG16 and transfer learning (Simonyan and Zisserman 2014). Numbers indicate the percentage of correctly identified images in the predefined test set, which was not used during training.

The main purpose of the current article is to explore the extent to which CNN feature transfer can be used in developing accurate diagnostic tools given realistic-size image sets and computational budgets available to systematists. The article represents one of the first applications of CNN feature transfer to challenging and realistic taxonomic tasks, where a high level of identification accuracy is expected. In contrast to previous studies, all independent identifications used here for training and validation have been provided by taxonomic experts with access to the imaged specimens. Thus, the experts have been able to examine characters that are critical for identification but that are not visible in the images, such as details of the ventral side of specimens imaged from above. The experts have also had access to collection data, which often facilitates identification.

We examined two types of challenging taxonomic tasks: 1) identification to higher groups when many specimens are likely to belong to subgroups that have not been seen previously and 2) identification of visually similar species that are difficult to separate even for experts. For the first task, we assembled two data sets consisting of diverse images of Diptera faces and the dorsal habitus of Coleoptera, respectively. For the second task, we used images of three closely related species of the Coleoptera genus *Oxythyrea*, and of nine species of Plecoptera larvae (Lytle et al. 2010). Training of the automated identification system was based entirely on the original images; no preprocessing was used to help the computer identify features significant for identification.

In all our experiments, we utilized the CNN architecture VGG16 with weights pretrained on the ImageNet data set (Simonyan and Zisserman 2014) for feature extraction, and a linear SVM (Cortes and Vapnik 1995) for classification. Our work focused on optimizing feature extraction techniques to reach high levels of identification accuracy. We also analyzed the errors made by the automated identification system in order to understand the limitations of our approach. Finally, to validate the generality of our findings, we tested our optimized system on several other biological image classification tasks studied in the recent literature on automated identification.

## Materials and Methods

### Data Sets and Baselines

**Figure 2.**
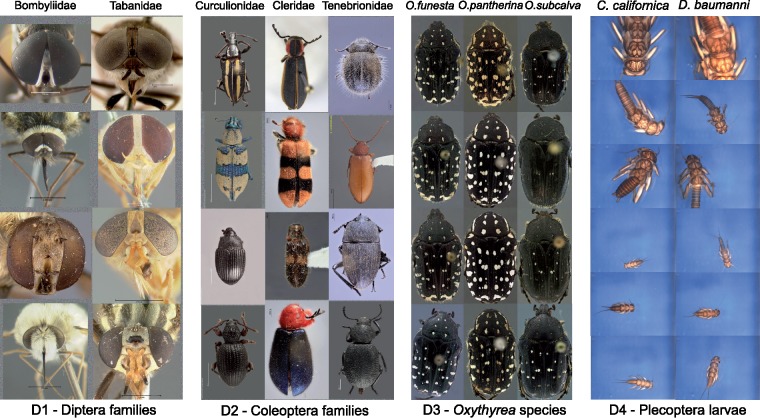
Sample images of each of the four data sets. For each data set, we show two or three different image categories. The example images illustrate some of the taxon diversity and variation in image capture settings within each category.

**Data sets**. We used four image data sets (D1–D4) for this study (Table 2, Fig. 2, Supplementary Material available on Dryad at http://dx.doi.org/10.5061/dryad.20ch6p5. The first two data sets (D1–D2) consist of taxonomically diverse images that were used in testing whether the automated system could learn to correctly circumscribe diverse higher groups based on exemplar images, and then use that knowledge in classifying images of previously unseen subtaxa of these groups. Data set D1 contains 884 images of Diptera faces, representing 884 unique species, 231 genera, and 11 families. Data set D2 has 2936 images of the dorsal habitus of Coleoptera, all belonging to unique specimens that together represent $CDATA[$CDATA[$>$$1932 species, $CDATA[$CDATA[$>$$593 genera, and 14 families (352 images identified only to genus and 339 only to tribe or family). Both D1 and D2 were used to test family-level identification. Subsets of D2 were also used to test discriminative power at the tribal level (data set D2A consisting of 21 tribes of Curculionidae with 14 or more images each (average 32); in total 675 images belonging to 109 unique genera) and at the genus level (data set D2B consisting of images of two genera, *Diplotaxis* [100 images] and *Phyllophaga* [121 images], both from the tribe Melolonthini of the family Scarabaeidae; in total, D2B contains images of 132 unique species; in two-thirds of the cases the images represent a single example of the species or one of two examples). D1 and D2 were assembled from images downloaded from iDigBio (http://idigbio.org) as described in the Supplementary Material available on Dryad. The identifications provided by iDigBio were assumed to be correct (but see Results for D2).

**Table 2.** Data sets used in this study

				Images per taxon	Image size		
Data set	Taxon	Level	No. of taxa	(range)	($CDATA[$CDATA[${\rm height} \times {\rm width}$$)	Part of insect	Source
D1	Flies	family	11	24–159	$CDATA[$CDATA[$946 \times 900$$	Face—frontal view	www.idigbio.org
D2	Beetles	family	14	18–900	$CDATA[$CDATA[$1811 \times 1187 $$	Body—dorsal view	www.idigbio.org
D3	Beetles	species	3	40–205	$CDATA[$CDATA[$ 633 \times 404$$	Body—dorsal view	this study
D4	Stoneflies	species	9	107–505	$CDATA[$CDATA[$960 \times 1280$$	Body—variable view	Lytle et al. 2010

The last two data sets (D3–D4) were used to test the success of the automated system in discriminating between visually similar species that are difficult to separate even for human experts. Data set D3 is original to this study; it consists of 339 images of the dorsal habitus of three closely related species (Sabatinelli 1981) of the genus *Oxythyrea*: *O. funesta*, *O. pantherina,* and *O. subcalva* (Coleoptera: Scarabaeidae). The depicted specimens are from four different countries of the south-west Mediterranean region where these taxa occur. Only *O. funesta* has a wider distribution, extending into Europe and Asia as well. One of the authors of the current article (DV) worked extensively on the taxonomy of this genus, based both on morphological and genetic data (Vondráček 2011; Vondráček et al. 2017). Current knowledge does not allow specimens of the three species studied here from North African localities to be identified with 100 % accuracy based only on the dorsal view of adults. Additional information from the ventral side of the specimen is also needed; the shape of the genitalia and the exact collection locality are also helpful in identifying problematic specimens (Sabatinelli 1981; Mikšić 1982; Baraud 1992). For D3, the identifications provided by DV after examination of the imaged specimens, and the locality data were assumed to be correct. Morphological species concepts were independently validated by genetic sequencing of other specimens.

Data set D4 was taken from a previous study of automated identification systems (Lytle et al. 2010) and consists of 3845 images of nine species of Plecoptera larvae representing nine different genera and seven families. The identification system developed in the original study (Lytle et al. 2010) was based on various handcrafted techniques to find image features of interest, such as corners, edges, and outlines. These features were then described with scale-invariant feature transform (SIFT) descriptors (Lowe 2004), which were subsequently submitted to a random forest classifier (Breiman 2001).

In contrast to the other data sets, the specimens of D4 are not portrayed in a standardized way. The position and orientation of the specimens vary randomly, and specimens may be damaged (missing legs or antennae). All larval stages are included, and the specimens vary dramatically in size. On one extreme, the specimen may be so small that it only occupies a small part of the image; on the other extreme, only part of the specimen may be visible (Lytle et al. 2010). The variation in D4 makes more information about each category available, which should make the identification engine more reliable and robust. On the other hand, different object sizes and viewing angles may require the system to learn a wider range of distinguishing features, making the identification task more challenging. It is not obvious which of these effects will be stronger.

Even though all species in D4 belong to different genera, two of the species (*Calineuria californica* and *Doroneuria baumanni*) are very similar and quite challenging to separate. The original study reported the identification accuracy achieved by humans, including some entomologists, in separating sample images of these two species after having been trained on separating them using 50 images of each species. We compared the human performance to the performance of our method in separating the same two species in the entire data set, which contains 490 images of *Calineuria* and 532 of *Doroneuria*). The true identifications provided in the original study were based on examination of the actual specimens by two independent taxonomic experts, which had to agree on the determination; these identifications are assumed here to be correct.

**Image characteristics**. The images in D1-D3 represent standardized views, in which specimens are oriented in a similar way and occupy a large portion of the image area. However, the exact orientation and position of appendages vary slightly, and some of the imaged specimens are partly damaged. In D4, both specimen orientation and size vary considerably as described above (Fig. 2). Images from all data sets were acquired in lab settings. Imaging conditions vary significantly in D1 and D2, since these images have different origins, but are standardized in D3 and D4. The backgrounds are generally uniform, particularly in D3 and D4, but the color is often manipulated in D1–D2 to get better contrast. In D2, some images have been manipulated to remove the background completely. Noise may be present both in the background (collection and determination labels, scales, measurements, glue cards, etc.) or on the specimens (pins, pin verdigris, dust etc.).

**Data preprocessing**. All three color channels (red, green, and blue) of the images were used. Before feeding images into CNNs, they are commonly resized to squares with VGG16 having default input size of $CDATA[$CDATA[$224\times224$$ pixels. Since D1, D2 and D3 images represent standard views but vary in image aspect ratio (height vs. width), resizing them to squares could lead to uneven distortion of objects from different categories, potentially resulting in information loss, or introducing bias that could interfere with training. Therefore, we performed the main experiments using images where the aspect ratio (height vs. width) was preserved. The best performing model was subsequently tested also on images that were resized to squares, even if it affected the aspect ratio.

To preserve aspect ratio, we first computed the average image height and width for each data set. Then we resized images such that one dimension of the image would fit the average size, and the other would be less than or equal to the average. If the latter was less than the average, we would center the image and then add random pixel values across all channels around the original image to fill the square frame. To preserve a minimalistic approach, no segmentation (cropping of the image) was performed, and the background was kept on all images.

**Training and validation sets**. We divided each data set into ten subsets using stratified random sampling, ensuring that the proportion of images belonging to each category was the same in all subsets. We then applied our procedure to the images belonging to nine of the subsets and used the last subset as the test image set. The reported accuracy values represent averages across ten repetitions of this procedure, each one using a different subset as the test set.

The images of D1–D3 all represent unique individuals, but D4 contains multiple images (4–5) of each specimen, varying in zooming level, pose, and orientation of the specimen (Lytle et al. 2010). The main results we report here do not take this into account; thus, the test set was likely to contain images of specimens that had already been seen during training. To investigate whether this affected the overall performance of our method, we repeated the procedure with a new partitioning of D4 into subsets in which all images of the same specimen were kept in the same subset, thus ensuring that none of the specimens imaged in the training set had been seen previously by the system.

### Model

We used VGG16 for feature extraction. VGG16 is known for its generalization capabilities and is commonly used as a base model for feature extraction (Caruana 1995; Bengio 2011; Yosinski et al. 2014). VGG16 has a simpler structure compared to other current architectures, such as Microsofts ResNets (He et al. 2016) or Google’s Inception v3 (Szegedy et al. 2016b), Inception v4 (Szegedy et al. 2016a), and Xception (Chollet 2016). However, these more complex models also tend to learn more domain-specific features, which makes them more challenging to use for feature extraction on unrelated tasks.

**Figure 3.**
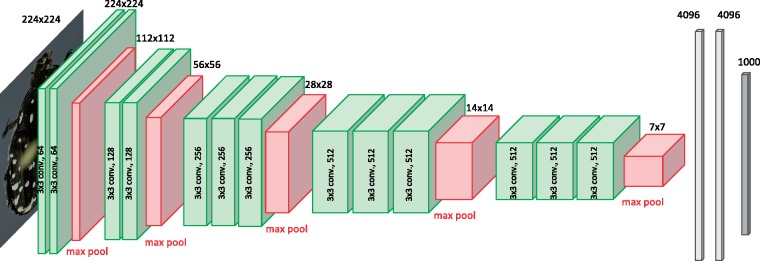
CNN model architecture. We used the CNN model VGG16 (Simonyan and Zisserman 2014) for feature extraction. The model consists of five convolutional blocks, each block consisting of two or three convolutional layers (green) followed by a MaxPooling layer (red). These blocks are followed by three layers of fully connected neurons (gray), the last of which consists of a vector of length 1000. Each element in this vector corresponds to a unique category in the ImageNet Challenge. The convolutional layers are obtained by applying different filters of size $CDATA[$CDATA[$3\times3$$ to the input from the preceding layer. The MaxPooling layer reduces the dimensions of the output matrix, known as the feature matrix, to half the width and half the height using spatial pooling, taking the maximum of the input values.

Specifically, we used VGG16 (Simonyan and Zisserman 2014) implemented in keras (Chollet 2015) with a TensorFlow backend (Abadi et al. 2016). In VGG16, the input images are processed in five convolutional blocks that we will refer to as *c1*– *c5*, respectively (Fig. 3). A block is made up of two or three convolutional layers, followed by a MaxPooling layer. Each convolutional layer applies a number of $CDATA[$CDATA[$3\times3$$ filters to the input. The number of different filters used in the convolutional layers vary across blocks: in the original VGG16 architecture used here, they are 64, 128, 256, 512, and 512 for convolutional block *c1*-*c5*, respectively. In the MaxPooling layer, the matrix is reduced to half the width and half the height by spatial pooling, taking the maximum value across a $CDATA[$CDATA[$2\times2$$ filter. Thus, an original image of $CDATA[$CDATA[$224\times224$$ pixels (the standard size used by VGG16) is reduced to an output matrix of height $CDATA[$CDATA[$\times$$ width $CDATA[$CDATA[$7\times7$$ ($CDATA[$CDATA[$224/2^5$$=7) after having passed through all five blocks. However, in the process the matrix has gained in depth through the application of filters in each of the convolutional layers. Thus, in block *c5* the total output dimension is $CDATA[$CDATA[$7\times7\times512$$, where 512 is the number of filters in this block. The output of each block is known as its *feature matrix*, and the feature matrix of *c5* is known as the *bottleneck features*. The last convolutional block is succeeded by three fully connected layers, the last of which provides the output related to the ImageNet classification task (Fig. 3).

### Feature Extraction

When using a CNN for feature extraction, it is common to focus on the fully connected final layers or on the bottleneck features, but feature matrices from deeper layers can also be used. It is standard practice to apply global pooling when extracting information from a feature matrix (Lin et al. 2013). Global pooling will reduce a feature matrix of dimension HxWxF—where H is height, W is width, and F is the number of filters—to a matrix of dimension $CDATA[$CDATA[$1\times1\times{\rm F}$$, that is, a vector of length F. This may result in information loss, but some reduction in dimensionality is needed, especially when using deeper feature matrices or when working with large input images. For instance, an image of size $CDATA[$CDATA[$416\times416$$ would produce a feature matrix of dimension $CDATA[$CDATA[$52\times52\times256$$ in block *c3* of VGG16, corresponding to almost 700,000 features; this would be very difficult to process for a classifier. Global pooling would reduce this feature matrix to a more manageable vector of just 256 feature elements. To optimize transfer learning for the taxonomic identification tasks we examined, we experimented with different methods of feature extraction and pooling as described below.

**Impact of image size and pooling strategy**. To examine the impact of image size and pooling strategy, we focused on bottleneck features. We analyzed the performance across all four data sets using input images being 128, 224, 320, 416, or 512 pixels wide, and using two different pooling strategies: global max pooling (using the maximum value for each filter layer) and global average pooling (using the average value). There are other dimensionality reduction strategies that could have been used, but these two pooling strategies completely dominate current CNN applications.

**Impact of features from different layers, multilayer feature fusion and normalization.** Using the optimal image size and pooling strategy from the previous step, we compared the discriminative potential of features from deeper convolutional blocks (*c1*, *c2*, *c3*, *c4*) with that of the commonly used bottleneck features of *c5*. We did not attempt to extract features from the final fully connected layers, as these top layers tend to learn high-level semantics specific for the task the model is trained on. In the ImageNet challenge, such semantics might include the presence or absence of typical cat or dog features, for example. Therefore, one might expect that features extracted from deeper layers of the CNN would give better results when the model is used as a feature extractor for unrelated classification tasks (Zheng et al. 2016). This should be particularly true for tasks where one attempts to separate visually similar categories, so-called *fine-grained* tasks, such as the taxonomic tasks we focus on in this article.

We also investigated the effect of feature fusion, introduced in Zheng et al. (2016); it involves concatenating feature vectors from several convolutional blocks. Finally, we examined the effect of signed square root normalization and 12-normalization as introduced in Arandjelović and Zisserman (2013). Normalization procedures are used in this context to reduce the variance of the elements in the feature vector. Reducing the influence of extreme values is known to improve the performance of SVMs.

**Impact of preserving more features.** Previous research has shown that globally pooled features generally give better performance than raw features in feature extraction (Zheng et al. 2016). However, it is not known whether intermediate levels of dimensionality reduction might give even better performance than either of these two extremes. To investigate this, we applied intermediate levels of pooling to the feature matrices from the three best performing convolutional blocks identified in the previous step.

Input images of the size selected in the first step, $CDATA[$CDATA[$416\times416$$, generate feature matrices of dimensions $CDATA[$CDATA[$52\times52\times256$$, $CDATA[$CDATA[$26\times26\times512$$, and $CDATA[$CDATA[$13\times13\times512$$ for *c3*, *c4*, and *c5*, respectively. If all features from the output matrix of *c4*, say, were used, a total of $CDATA[$CDATA[$26\times26\times512$$ = 346,112 features would be obtained. In contrast, global pooling would yield a 1D vector of only $CDATA[$CDATA[$1\times1\times512$$ = 512 features. To explore intermediate levels of data compression, we applied pooling of various degrees to obtain NxNxF feature matrices, each of which was then reshaped and flattened to a 1D feature vector. To standardize the dimensionality reduction procedure, we first generated $CDATA[$CDATA[$26\times26\times F$$ feature matrices for each of *c3*, *c4*, and *c5* as follows. For *c3*, the output feature matrix of size $CDATA[$CDATA[$52\times52\times256$$ was average-pooled to $CDATA[$CDATA[$26\times26\times256$$ applying a $CDATA[$CDATA[$2\times2$$ filter with stride two. The features of *c4* already had the correct dimension ($CDATA[$CDATA[$26\times26\times512$$), while the features of *c5* were extracted before the last MaxPooling layer to retain the input size $CDATA[$CDATA[$26\times26\times512$$ of *c5*.

To each of these $CDATA[$CDATA[$26\times26\times F$$ feature matrices, we then added zero-padding to get feature matrices of size $CDATA[$CDATA[$28\times28\times F$$. Using spatial pooling of various degrees, the matrices were then reduced to matrices of size $CDATA[$CDATA[$14\times14\times F$$, $CDATA[$CDATA[$7\times7\times F$$, $CDATA[$CDATA[$4\times4\times F$$, and $CDATA[$CDATA[$2\times2\times F$$. Each of these matrices was reshaped and flattened to a one-dimensional feature vector, and its performance compared that of the $CDATA[$CDATA[$1\times1\times F$$ feature vector resulting from global pooling. We also investigated the effect of combining intermediate-level pooling with feature fusion for *c3*+*c4*, *c3*+*c5*, *c4*+*c5*, and *c3*+*c4*+*c5*. Before training the classifier on the data, we applied signed square root normalization.

### Classifier and Evaluation of Identification performance

**Classifier**. As a classifier for the extracted features, we used an SVM method (Cortes and Vapnik 1995), which is a common choice for these applications (Donahue et al. 2014; Oquab et al. 2014; Razavian et al. 2014; Zeiler and Fergus 2014; Azizpour et al. 2016; Zheng et al. 2016). SVM performance is at least on par with other methods that have been studied so far (e.g., Azizpour et al. 2016). SVMs are supervised learning models that map inputs to points in high-dimensional space in such a way that the separate categories are divided by gaps that are as wide and clear as possible. Once the SVM classifier is trained, new examples are mapped into the same space and predicted to belong to a category based on which side of the gaps they fall.

SVMs are memory efficient because they only use a subset of training points (support vectors). They generalize well to high dimensional spaces (Vapnik and Chapelle 2000) and are suitable for small data sets where the number of dimensions is greater than the number of examples (Vapnik 1999). SVMs are also good at learning from imbalanced data sets (He and Garcia 2009), even though the performance tends to be lower on categories with few examples.

Specifically, we used the linear support vector classification algorithm (LinearSVC) implemented in the scikit-learn Python package (Pedregosa et al. 2011) under default settings. LinearSVC uses a one-versus-all strategy, which scales well (linearly) with the number of categories. Other SVM implementations use a one-versus-one scheme, the time complexity of which increases with the square of the number of categories. A disadvantage of LinearSVC is that it does not provide probability estimates.

**Measuring identification performance**. The identification performance was tested on the four main data sets and the data subsets described above. All accuracies reported were calculated as the proportion of predicted labels (identifications) that exactly match the corresponding true labels. Error rate (or misclassification rate) is the complement of accuracy, that is, the proportion of predicted labels that do not match the true labels.

**Impact of sample size**. To examine the influence of the image sample on identification performance, we studied the relation between the identification accuracy for each category and the number and types of images of that category in D1 and D2. We also created random subsets of D4 with smaller number of images per species than the original data set.

**Recently published image identification challenges**. To validate the generality of our findings, we explored the performance of our approach on several recently published biological image data sets used to develop automated identification systems for tasks similar to the ones studied here. We did not optimize a model for each of these data sets; instead, we used a standard model that performed well across data sets D1–D4 according to the results from our optimization experiments.

Specifically, we used the following five data sets to test the general performance of our standard model: 1) *ClearedLeaf*, consisting of 7597 images of cleared leaves from 6949 species and 2001 genera (Wilf et al. 2016); 2) *CRLeaves*, containing 7262 leaf scans of 255 species of plants from Costa Rica (Mata-Montero and Carranza-Rojas 2015); 3) *EcuadorMoths*, containing 2120 images of 675 moth species, all genetically verified but only some properly morphologically studied and described, belonging to the family Geometridae (Brehm et al. 2013); 4) *Flavia*, with 1907 images of leaves from 32 plant species (Wu et al. 2007); and 5) *Pollen23*, covering the pollen grains of 23 Brazilian plant species (805 images in total) (Gonçalves et al. 2016). For ClearedLeaf, we used one of the identification challenges in the original study, involving families with 100+ images each.

**Scripting and visualization**. The python scripts used for the experiments are provided as Supplementary Material available on Dryad. Visualizations were generated using the plotly package (Plotly Technologies 2015). Image feature space was visualized using t-distributed stochastic neighbor embedding (t-SNE) (Maaten and Hinton 2008), a popular technique for exploring high dimensional data in 2D or 3D space (Fig. 12). It is an unsupervised method where the identities of the images are not provided during training. The identities are only used subsequently to assign different colors to the data points.

## Results

### Feature Extraction

**Figure 4.**
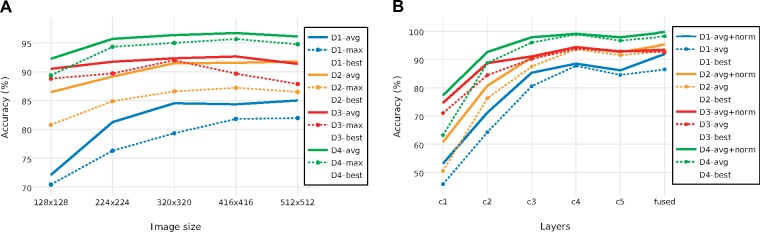
a) Impact of image size and average versus max global pooling of *c5* features on identification accuracy. Data sets are separated by color, and the best result for each data set is indicated with a cross (X). Global average pooling was always better than global max pooling. In general, $CDATA[$CDATA[$416\times416$$ tended to be the best image size. It was the optimal size for two out of four data sets (global average pooling) or three out of four data sets (global max pooling). b) Impact of feature depth, normalization and feature fusion on identification accuracy. Input images of size $CDATA[$CDATA[$416\times416$$ and average global pooling was used in all cases. Data set colors and indication of the settings giving the best identification performance as in (a); note that the performance of feature fusion here is slightly different from that in (a) because of minor differences in the protocol between the experiments. Normalization and feature fusion generally improved identification accuracy, although *c4* features tended to outperform features from all other individual layers.

**Impact of image size and pooling strategy**. Our results showed that identification performance was significantly affected by image size and pooling strategy (Fig. 4). The use of global average pooling yielded notably better results than global max pooling (Fig. 4a). This may be related to the fact that the imaged specimens in our data sets typically occupy a large fraction of the image, such that features across the entire image contribute to identification. The difference between average pooling and max pooling was slightly smaller in D4, which might be explained by this data set containing a substantial number of images of small specimens occupying only a tiny part of the picture frame.

Input images of size $CDATA[$CDATA[$416\times416$$ performed better than other image sizes when global max pooling was used, with the exception of D3 where $CDATA[$CDATA[$320\times320$$ images were best. When global average pooling was used instead, the optimal image size was still $CDATA[$CDATA[$416\times416$$ for D3 and D4, but the largest image size ($CDATA[$CDATA[$512\times512$$) yielded a slight improvement over $CDATA[$CDATA[$416\times416$$ images for D1 and D2. Based on these results, we decided to proceed with the remaining optimization steps using input images of size $CDATA[$CDATA[$416\times416$$ and global average pooling, unless noted otherwise.

**Impact of features from different layers, multilayer feature fusion, and normalization**. Using *c4* features resulted in the best identification results across all data sets (Fig. 4b). Features from *c4* consistently outperformed the features from *c5*; sometimes, *c5* features were also outperformed by *c3* features. In D3, high identification accuracy was already obtained with features from *c2*. This might be due to the fact that the classes in D3, three sibling species, are differentiated by minute details (quantity, location, and size of the white spots on the elytra and on the pronotum, and the amount of setae), which are presumably well represented by feature matrices close to the raw image. Signed square root normalization consistently improved identification accuracy (Fig. 4b), while adding 12-normalization deteriorated performance.

The fused features generated better identification results than features from individual convolutional blocks in all data sets except D3, where the *c4* features alone were slightly better than fused features (5.57% against 6.48% error rate). Feature fusion was particularly effective for D1, where the error rate was reduced by one-third over that of *c4* features alone (from 11.43% to 8%). The effect on D2 was also significant (a reduction by 14%, from 5.48% to 4.73% error rate). Even for D4, where identification accuracies were generally very high, feature fusion resulted in a significant reduction in error rate (from 0.86% to 0.34%).

Using global max pooling instead of global average pooling resulted in reduced identification accuracy, but otherwise gave similar results with respect to the relative performances of both individual and fused feature matrices. Combining features from global average and global max pooling did not improve the results; instead, the identification performance was intermediate between that of each method applied separately (Supplementary Fig. 1).

**Figure 5.**
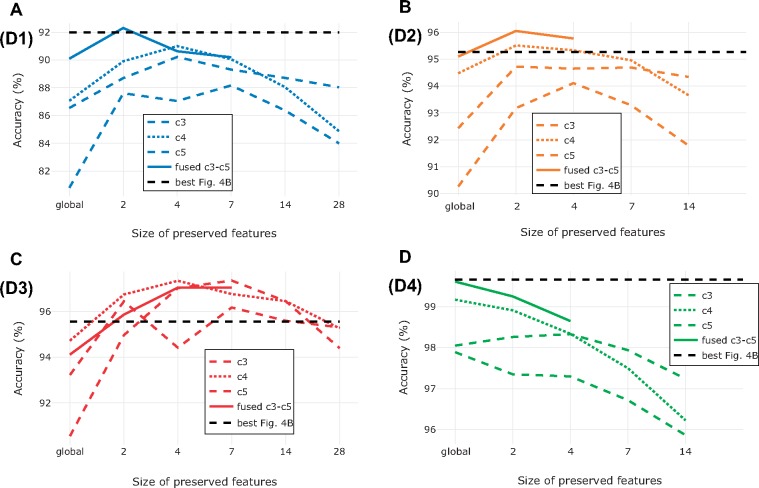
Impact of preserving more features during feature extraction on identification accuracy for data sets D1 (a), D2 (b), D3 (c), and D4 (d). We used input images of size $CDATA[$CDATA[$416\times416$$ in all cases, and we pooled features from each of the last three convolutional blocks (*c3*–*c5*) into matrices of dimension $CDATA[$CDATA[${\rm N}\times{\rm N}\times{\rm F}$$, where F is the number of filters of the corresponding convolutional block and N was set to 1 (global average pooling), 2, 4, 7, 14, or 28. The dashed black line indicates the best performing model from previous experiments (Fig. 4b). For D2 and D4 we stopped at N = 14 because of the computational cost involved in computing the value for N = 28, and since the accuracy had already dropped significantly at N = 14. Because of the computational cost, we computed performance of fused feature matrices only up to N = 7 (D1 and D3) or N = 4 (D2 and D4). On data sets D1, D2, and D3, the optimum identification performance was somewhere between globally pooled and nonpooled features. For D4, global pooling yielded the best identification performance.

**Impact of preserving more features**. For three out of four data sets, the best results were not obtained with fusion of globally pooled features (Fig. 5). For D1–D3, preserving more features ($CDATA[$CDATA[$2\times2\times{\rm F}$$, $CDATA[$CDATA[$4\times4\times{\rm F}$$ or $CDATA[$CDATA[$7\times7\times{\rm F}$$) from the best performing convolutional block yielded identification results that were competitive with those from fused, globally pooled features ($CDATA[$CDATA[$1\times1\times{\rm F}$$). In the case of D3, the nonglobal features from *c3*, *c4,* and *c5* all outperformed the best result from the previous experiments, cutting the error rate in half (from 5.57% to 2.64%). It is also interesting to note that *c3* achieved identification accuracies that were comparable to those of *c4* when more fine-grained feature information was retained. An explanation for this result could be that differences between the three sibling species in D3 are due to small details represented by only a few pixels each, information that could easily be lost in dimensionality reduction.

**Figure 6.**
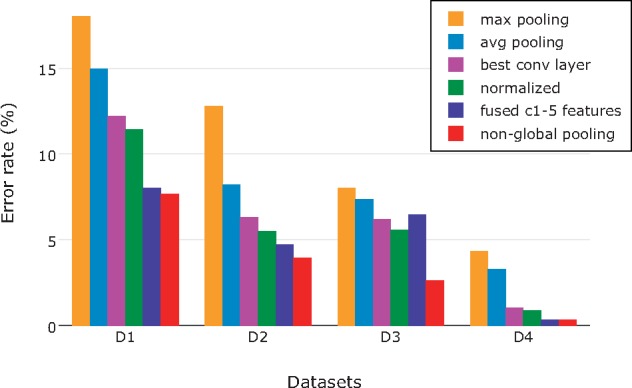
Reduction in error rate throughout our optimization steps, using input images of size $CDATA[$CDATA[$416\times416$$. The minimum error rates (and top identification accuracies) for D1–D3 were achieved after applying nonglobal feature pooling in the last step (red bar). Note the drastic improvement that came with nonglobal feature pooling in D3, the most fine-grained data set studied here. The best result for D4 was achieved already in the penultimate step, by fusing globally pooled features from *c1* to *c5*.

**Impact of aspect ratio**. All results reported above were obtained with images where the aspect ratio was maintained, even if it involved introduction of uninformative pixels to fill out the square input frame typically used with VGG16. To inspect how this affects identification performance, we exposed the best performing models to images where the input frame had been filled completely even if that distorted the proportions of the imaged objects. The performance was similar to that in the original experiments, with only slight changes in accuracy (+0.38%, +0.27%, +0.77%, and +0.03% for D1–D4, respectively).

### Taxonomic Identification

#### Task 1—Identifying Insects to Higher Groups

**Figure 7.**
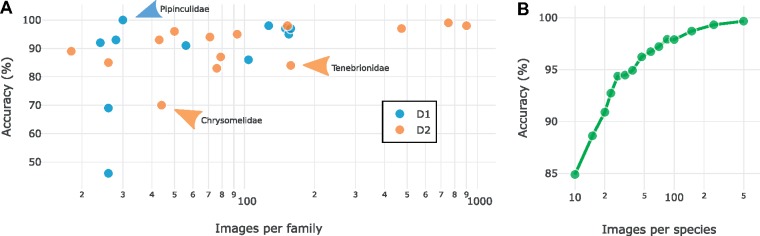
Effect of sample size on identification accuracy. a) Scatter plot of identification accuracies against sample sizes for all families from D1 and D2. Note that accuracies varied more across families at small sample sizes. Accuracies were also affected by how well the sample covered the variation in each family; such effects appear to explain many of the outliers (arrows; see text). b) The effect of the number of images per species on the performance of the best model on D4. Each point represents the average across ten random subsamples of the original data set.

**Data set D1. Frontal view of the head of Diptera specimens**. The best model achieved 7.35% error rate when discriminating between the 11 families in D1 (Fig. 9). As expected, families represented by few specimens were more difficult for the model to identify correctly than those represented by many exemplars (Fig. 7). Interestingly, the Pipunculidae were the most easily identified family, even though they were only represented by 30 images. The vernacular name of this family is big-headed flies, referring to the big eyes that cover nearly the entire head. This highly distinctive feature makes it particularly easy to identify members of this family from head images. Among the misclassified images, the second best guess of the model was correct in approximately two out of three cases (Fig. 9), indicating that the model was usually not completely off even in these cases.

**Figure 8.**
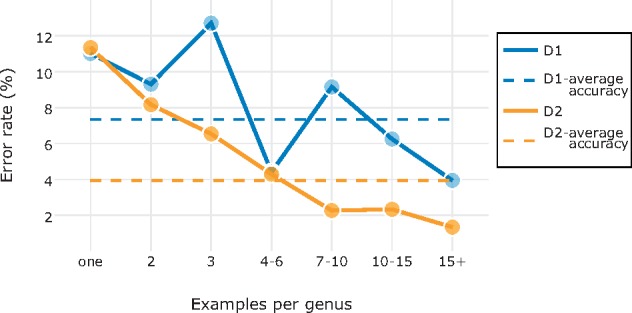
Effect of sample nature on identification accuracy. Scatter plot of identification accuracies against the number of example images per genus for all families from D1 and D2. The more examples there were on average of each genus in the family, the more likely it was that the model had seen something similar to the image being classified, lowering the misclassification rate.

Almost one-third (29%) of D1 images are from genera represented by only one, two, or three examples. The model was able to learn from at most two examples from such a genus, and often none. As one might expect, our findings showed that the families with a small average number of exemplars per genus were also the ones that were most difficult for the model to learn to identify correctly (see Fig. 8).

**Figure 9.**
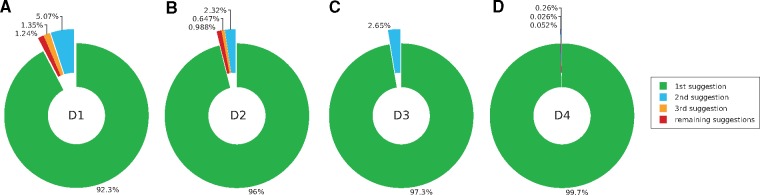
Proportion of correct identifications from the top three suggestions of the model for data set D1 (a), D2 (b), D3 (c), and D4 (d). When the most probable identification (first suggestion) of the model was erroneous, the second most probable identification was usually correct, indicating that the model was not too far off.

**Data set D2—Families of Coleoptera**. The best model had an error rate of 3.95$CDATA[$CDATA[$\%$$ (116/2936) when discriminating between the 14 beetle families in D2 (Fig. 9). As for D1, the number of examples per category affected the result significantly (Fig. 7), with more examples giving higher identification accuracy. A notable outlier is the family Tenebrionidae, which had an unexpectedly low identification accuracy (85%) given the sample size (158 images). Tenebrionidae is a cosmopolitan and highly diverse family with over 20,000 species. Tenebrionids vary significantly in shape, color and morphological features, and the family is well known to contain species that superficially look similar to typical representatives of other families of Coleoptera.

Another case that stands out is the family Chrysomelidae (70% accuracy) (Fig. 7). The poor identification performance for this family was likely related to the small number of examples (44 images), and the fact that these examples covered a significant amount of the morphological diversity in the family (37,000 species described to date). The Chrysomelidae examples for which we had at least genus name (40 of 44 images) are scattered over 25 genera, 11 tribes, and 7 subfamilies. Interestingly, one of the subfamilies is better represented than the others, the Cassidinae (14 examples from 10 genera and 3 tribes); only one of these images was misclassified by the best model.

As for D2, the most challenging images were those belonging to genera likely to have few or no examples in the training set (Fig. 8). For the majority of the misclassified images, the second best guess of the model was correct (Fig. 9).

After we had completed the experiments, we discovered that seven of the D2 images had been erroneously classified by the experts submitting them to iDigBio. This means that the training set we used was not clean but contained 0.2% noise on average. Apparently, this did not seem to be a significant obstacle in developing a model with high identification performance. When the erroneously labeled images were in the test set, the best model identified four of them to the right family. Of the remaining three specimens, one does not belong to any of the 14 families studied here, and another one is a poorly preserved specimen for which we were not able to establish the correct family identification ourselves with any degree of confidence.

**Data set D2A—Tribes of Curculionidae**. Applying the best model for D2 on this task, we reached an overall identification accuracy of $CDATA[$CDATA[$>$$79%. The tribes with fewer examples were generally more challenging to identify correctly. The accuracy dropped to 42% when no example of the genus was included in the training set, and increased to more than 90% for genera with $CDATA[$CDATA[$>$$15 examples.

**D2B—Genera of Melolonthini**. The best model for D2 was able to separate the two chosen genera of Melolonthini with an error rate of 2.7%. The misclassified images were predominantly from species that had not been seen by the model (5 of 6 misclassified images were the only exemplar of the species). For species with more than three example images, the error rate dropped to 1.6%, compared to 3.2% for species represented by only one example image.

The two genera in D2B are from different subtribes, and experts can easily distinguish them by looking at features of the abdomen and the labrum. However, none of these characters is visible in images of the dorsal habitus, making the task of identifying the genera from the D2B images quite challenging even for experts. We asked one of the leading experts on Melolonthini, Aleš Bezděk, to identify the images in D2B to genus; his error rate was 4.1%.

### Task 2—Identifying Visually Similar Species

**D3—Closely related species of *Oxythyrea***. The best model had a misclassification rate of only 2.67%. *Oxythyrea funesta* was the most challenging species to identify, with five misclassified images (5.2% error rate), followed by *O. pantherina* with three misclassified images (1.5% error rate) and *O. subcalva* with one misclassified image (2.5% error rate).

**Figure 10.**
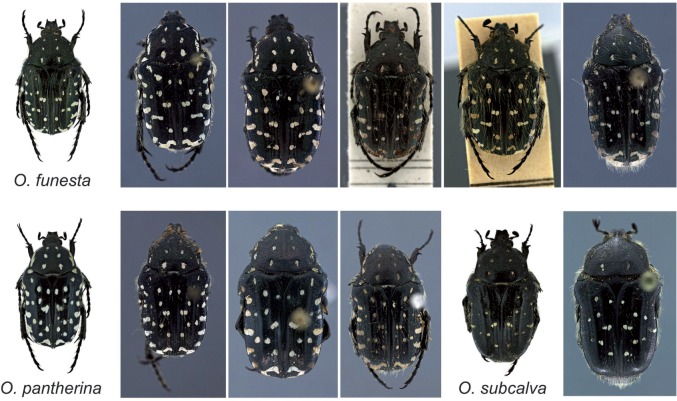
Examples of misclassified images from D3 (*Oxythyrea*). Next to the misclassified images of *O. funesta* (5 images), *O. pantherina* (3 images), and *O. subcalva* (1 image) we show typical representatives of the three *Oxythyrea* species. Note, for instance, that the first two misclassified specimens of *O. funesta* are quite similar to typical specimens of *O. pantherina*, the identification suggested by our model.

Some of the misclassified images are indeed challenging (Fig. 10). For instance, the left two misclassified specimens of *O. funesta* would probably be identified by most humans as *O. pantherina*, the species that our model suggested. Similarly, all three misclassified specimens of *O. pantherina* are more similar to *O. funesta*, the identification suggested by our model, than to typical *O. pantherina*.

D.V., who studied the genus for 8 years, estimates that he can reliably identify up to 90% of the specimens of these three species based on dorsal habitus alone. When challenged with the D3 images, DV was able to identify 95% of the images correctly, but some of those identifications were aided by the fact that he recognized the imaged specimens and remembered what species he had identified them to when he photographed them.

**D4—Species of Plecoptera larvae**. The original study reported 5.5% error rate for this data set using a system based on handcrafted feature extraction (Lowe 2004). The best of our models had an error rate of only 0.34%. On four of the nine species it reached 100% accuracy, including *Moselia infuscata*, the species with the smallest number of examples and the highest misclassification rate in the original study (10.1%).

**Figure 11.**
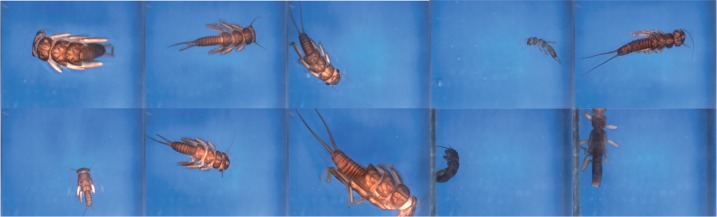
Examples of misclassified images from D4 (Plecoptera larvae). Note that most misclassified specimens are small or photographed from angles making them difficult to identify.

In the images misclassified by our model (Fig. 11), it is noticeable that the specimen is often turned toward the side, unlike the majority of the images where the specimen is seen from above. Many of the misclassified specimens are also small, occupying a small portion of the picture frame. However, even for the misclassified images, the model mostly (9 of 13 cases) suggested the correct taxonomic identification with the second highest probability (Fig. 9).

**Figure 12.**
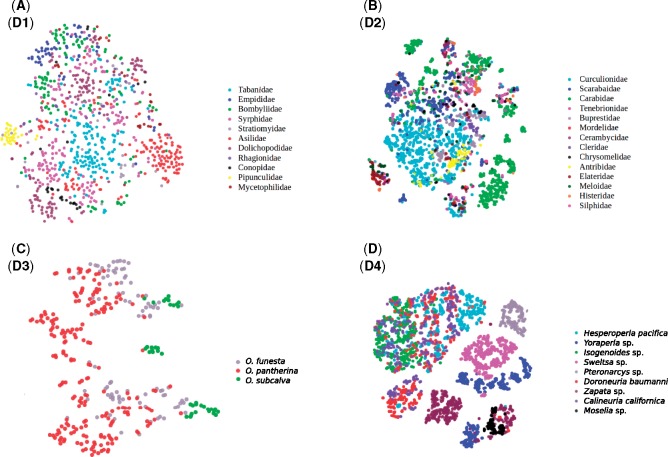
Visualization of the *c4* feature space of data sets D1 (a), D2 (b), D3 (c), and D4 (d) using t-SNE (Maaten and Hinton 2008). The *c4* features contain a considerable amount of information that is useful in separating image categories. Note that some categories are more isolated and easier to identify than others. Note also that it is common for categories to consist of several disjunct sets of images. These disjunct sets are likely to represent images that differ from each other in features that are irrelevant for identification, such as background color or life stage.

Larios et al. (2008) trained 26 people, most of whom had some prior training in entomology, to distinguish *Calineuria californica* from *Doroneuria baumanni* (Fig. 2). Of the nine species, these are the ones that are most difficult to separate from each other. Participants were first trained on 50 random samples from each species, and then tested on another set of 50 images. The study reported an error rate of 22%.

These two species were also the ones that were most often confused by our model: all misclassified images of *C. californica* were classified as *D. baumanni*, and vice versa. However, our model misclassified only four images of *C. californica* (0.82% error rate) and four images of *D. baumanni* (0.75% error rate), even though it was faced with the harder task of classifying the images into one of nine species instead of just separating these two species. The original study reported misclassification rates of 6.7% and 5.1% for *C. californica* and *D. baumanni*, respectively.

D4 contains multiple images (4–5) of the same specimen, varying in zoom, pose, and orientation (Lytle et al. 2010). In the original experiment, we did not keep these images together. Thus, the test set would typically contain images of specimens that had already been seen during training, albeit in a different magnification, and seen from a different angle and in a different pose. When all images of the same specimen were kept in the same fold, such that test images never included specimens that had been seen before, the accuracy of the best model remained high (98.62% accuracy and corresponding to 1.38% error rate).

Under these more stringent conditions, the majority of misclassified images belonged to specimens that differ markedly from typical representatives of the species. For instance, one specimen of *Isogenoides* is much bigger and darker (probably representing a later larval stage) than the other specimens; three of the five images of this specimen were misclassified. One specimen of *Hesperoperla pacifica* is pale while others are dark (four of five images misclassified) and one *Zapata* specimen is completely curved and photographed from a lateral view, which is not the case for the other specimens (five of five images misclassified). Misclassified *C. californica* and *D. baumanni* have parts occluded, are photographed from a lateral view, are unusually curved, or are really small and pale. It is interesting to note that all but eight of the 774 imaged specimens (1%) had the majority of their images correctly identified by our model.

### Performance on Related Tasks

To examine the generality of our results, we tested the performance of our approach on five related biological image identification challenges that have been examined in recent studies of automated identification systems. We did not optimize our model for each of these data sets. Instead, we used settings that performed well across all of the data sets D1–D4. Specifically, unless noted otherwise, we used input images of size $CDATA[$CDATA[$416\times416$$, global average pooling, fusion of *c1*–*c5* features, and signed square root normalization.

**ClearedLeaF**. The original study (Wilf et al. 2016) reported results on various identification tasks involving this data set, using manual extraction of SIFT descriptors (see Lowe 2004). We randomly picked one of the tasks, identifying families with 100+ images each. The original study reported 72.14 % accuracy on this task; with our approach, we reached an identification accuracy of $CDATA[$CDATA[$>$$88%.

**CRLeaves**. Using fine-tuned Inception v3 (Szegedy et al. 2016b) pretrained on ImageNet, Rojas et al. (2017) report 51% accuracy in identifying the 7262 images of CRLeaves to the 255 included plant species. They also tried fine-tuning on a bigger plant data set (253,733 images) followed by fine-tuning on target tasks such as CRLeaves. Although this approach improved accuracies by $CDATA[$CDATA[$~$$5% on other data sets examined in their study, it actually lowered the performance on CRLeaves (49.1% accuracy). Our model achieved an identification accuracy of $CDATA[$CDATA[$>$$94%.

**EcuadorMoths**. This data set of 675 geometrid moth species (2120 images) is extremely challenging because of the visual similarity of the species and the small number of images per species. Previous studies have reported 55.7% accuracy (Rodner et al. 2015) and 57.19% accuracy (Wei et al. 2017). Both studies were based on models pretrained on ImageNet (Russakovsky et al. 2015). The first utilized feature extraction by globally pooling bottleneck features from AlexNet (Krizhevsky et al. 2012). The second utilized VGG16 (Simonyan and Zisserman 2014), introducing a novel approach the authors called selective convolutional descriptor aggregation (SCDA) for localizing the main object and removing noise. Wei et al. (2017) show that the SCDA approach outperforms 11 other CNN methods on EcuadorMoths. They used a training set of 1445 images, containing on average only two images per species. Using the same partitioning into training and test data set, our model achieved $CDATA[$CDATA[$~$$ 55.4 % identification accuracy, slightly worse than both previous studies. However, features from block *c4* alone outperformed the default settings (58.2%). This is similar to the results we found for D3, which is also a task with visually similar and closely related species.

**Flavia**. The original study used handcrafted feature extraction and reported 90.3% identification accuracy (Wu et al. 2007). This was subsequently improved with other traditional techniques (93.2 %, 97.2 %) (Kulkarni et al. 2013; Kadir 2014). The performance was recently pushed even further with deep belief networks (99.37% accuracy) (Liu and Kan 2016) and by several CNN-based approaches (97.9 % and 99.65% accuracy; Barré et al. 2014 and Sun et al. 2017, respectively). Our base model misidentified only one image in Flavia, achieving 99.95% accuracy.

**Pollen23.** The original study reported 64 % accuracy on this task with the best-performing model (Gonçalves et al. 2016). It was based on combination of handcrafted features, such as color, shape and texture, and a method known as “bag of words” (Yang et al. 2007). The performance was comparable to that of beekeepers, who were trained for this particular task; they achieved 67% accuracy. Images from this data set are small (approximately one half of the images having width or height $CDATA[$CDATA[$<$$224 pixels, the default input size for VGG16, so we decided to use $CDATA[$CDATA[$160\times160$$ as the size of our input images. Our model achieved $CDATA[$CDATA[$>$$94% identification accuracy on this task.

## Discussion

### CNN Feature Transfer

The ultimate goal of the machine learning community is to build systems that can perceive and execute highly complex tasks without prior knowledge and with minimal if any human intervention (Bengio and LeCun 2007). In recent years, DL and CNNs have contributed to spectacular advances in this direction in computer vision. Image classification tasks can now be solved with a very high degree of accuracy using systems that support end-to-end learning (LeCun et al. 2015).

In this study, our aim was to explore the extent to which systematists can build on these breakthroughs in developing automated taxonomic identification systems. Clearly, we currently lack both sufficiently large training sets and enough computational resources to train state-of-the-art CNNs from scratch on taxonomic identification tasks. Therefore, some form of transfer learning is needed, and feature transfer has recently emerged as the method of choice.

Perhaps, the most important reason for this is that feature transfer is more computationally efficient than fine-tuning. In fine-tuning, the images have to be run through the CNN in a forward pass, and then the computed derivatives from the predictions have to be backpropagated to modify the CNN, which is computationally expensive. Even worse, this process of alternating forward and backward passes has to be repeated until our model converges, which only happens if we have chosen the right parameters. Choosing the right parameters is not trivial; it requires machine learning knowledge. In feature extraction, the image is only passed forward through the pretrained CNN once; no backpropagation or fine-tuning of the model is needed. The only parameter tuning that is needed in feature extraction is the adaptation of the classifier, which is fast because the classifier focuses entirely on the smaller training set for the target task.

Feature transfer is known to outperform hand-engineered features on some tasks (Donahue et al. 2014) and fine-tuning often further helps the performance (Li and Hoiem 2016). However, when fine-tuning on small or on unbalanced data set, overfitting is a serious concern. This is usually tackled with aggressive data augmentation, which may include geometric (cropping, flipping, rotating, shifting, and distorting) or color (shading, shifting color channels, or changing the brightness) transformations. Both the original images and their duplicates ($CDATA[$CDATA[$D$$ instances) are then used in training the network, increasing the size of the training set from $CDATA[$CDATA[$N$$ to $CDATA[$CDATA[$N(D+1)$$ images. This further increases the computational cost of fine-tuning.

For these reasons, the combination of a pretrained CNN, usually VGG16, for feature extraction and linear SVMs for classification is a common choice in recent computer vision studies (Donahue et al. 2014; Razavian et al. 2014; Azizpour et al. 2016; Zheng et al. 2016). Nevertheless, our results show that there is still room for improvement. Specifically, by experimenting with the feature extraction strategy, our results show that the identification accuracy can be improved significantly compared to the basic off-the-shelf approach that involves global max pooling of bottleneck features from input images of size $CDATA[$CDATA[$224\times224$$ (Figs. 4–6).

Our finding that global average pooling performs better than global max pooling (Fig. 4a) is congruent with the results of a recent study applying feature transfer to fine-grained classification tasks (Zheng et al. 2016). A possible explanation for the better performance of average pooling is that the objects in our images tend to be big and occupy a large part of the picture frame. Thus, features from all parts of the image contribute to identification, which they can do through the average but not the max value. This explanation is consistent with the observation that the difference between average and max pooling was smaller for D4, containing a number of images of smaller objects (Fig. 4a).

Our results suggest that there may be an advantage of using intermediate-level features compared to bottleneck features or features from the final layers. These findings contrast with those from generic classification tasks, such as Caltech-101 (Fei-Fei et al. 2006), Caltech-256 (Griffin et al. 2007), and PASCAL VOC07 (Everingham et al. 2010), where the first fully connected layer has been found to be the most discriminative layer. It also differs from the results of previous studies of fine-grained identification tasks, such as Bird-200-2011 (Wah et al. 2011) and Flower-102 (Nilsback and Zisserman 2008), where it has been found that *c5* is the most discriminative layer (Zheng et al. 2016). One possible explanation for these differences is that our data sets are more fine-grained than any of the data sets examined in these studies. However, the results we obtained on other recently published data sets suggest that fusion of intermediate-level features will perform well on a broad range of biological image classification tasks.

Our results also indicate that preserving more features than the standard off-the-shelf approach may give better identification performance (Fig. 4b). Preserving more features increases the computational cost, but this may be a price worth paying when working with challenging fine-grained classification tasks, or when the identification accuracy is more important than the computational cost of training the classifier. The advantage of preserving more features is most evident on D3, which is the most fine-grained of all the data sets studied here (Fig. 6).

Recent research has shown that CNNs trained from scratch on the large ImageNet data set perform well despite the presence of small amounts of noise (0.3 %) (Russakovsky et al. 2015). Our results for D2 (0.2% noise) indicate that also feature transfer from pretrained CNNs is robust to a similar level of noise in the training set for the target task. This facilitates practical use of methods based on CNN feature transfer, as it can be exceedingly difficult to completely eliminate mislabeled data from training sets even when identifications are provided by experts.

It is somewhat surprising that the optimal feature extraction strategy turned out to be so similar across all tasks and data sets (D1–D4) examined in detail here, despite the fact that they are so varied. It is true that D1–D3 share several important features: the object comprises a major part of the image and the images are standardized with respect to viewing angle and orientation of the specimen. However, these features are not shared by D4. The tasks are also different in that D1–D2 involve the recognition of variable higher taxa, while D3–D4 involve the identification of more homogeneous categories with fine-grained differences among them.

The only striking features that are shared across all problems examined here are the small training sets, the clean images (few distracting objects), and the high labeling (and expected identification) accuracy. Thus, it might be possible to find a single feature extraction protocol that would perform well across a broad class of biological identification problems with similar properties. This prediction is supported by the results from applying a standard feature extraction protocol inspired by our results for D1–D4 on a range of recently studied biological image classification problems (Table 3). Without task-specific optimization, our approach managed to beat the state-of-the-art identification accuracy for almost all data sets, often with a substantial margin.

**Table 3.** Experiments on recently published biological image classification tasks

Data sets	Categories	Our method	Original study	Method	Reference
ClearedLeaf	19 families	**88.7**	71	SIFT + SVM	(Wilf et al., 2016)
CRLeaves	255 species	**94.67**	51	finetune InceptionV3	(Carranza-Rojas et al., 2017)
EcuadorMoths	675 species	55.4 (**58.2**)	55.7 57.19	AlexNet + SVM VGG16 + SCDA + SVM	(Rodner et al., 2015) (Wei et al., 2017)
Flavia	32 species	**99.95**	99.65	ResNet26	(Sun et al., 2017)
Pollen23	23 species	**94.8**	64	CST + BOW	(Gonçalves et al., 2016)

*Note*: We used input images of size $CDATA[$CDATA[$416\times416$$ ($CDATA[$CDATA[$160\times160$$ for Pollen23), global average pooling, fusion of *c1*–*c5* features, and signed square root normalization. Accuracy is reported using 10-fold cross validation except for EcuadorMoths, where we used the same partitioning as in the original study. Bold font indicates the best identification performance.

Undoubtedly, further work can help improve the performance of the method described here. For instance, preprocessing images by implementing object segmentation (separation of objects from the background) has been shown to improve the performance of image classification in general (Rabinovich et al. 2007), and it seems likely it will also help improve our CNN feature extraction protocol. Other interesting techniques that have emerged recently, and that should be tried in the CNN feature extraction framework, include: assembling multiple architectures as in bilinear CNNs (Lin et al. 2015); using multiple images as inputs as in Siamese CNNs (Zbonar and LeCun 2016); and identifying informative image parts with HSnet search prior to feeding them into the CNN (Lam et al. 2017).

It may also be possible to improve identification performance by rethinking taxonomic tasks. For instance, preliminary experiments that we performed indicate that identification performance can be increased by subdividing the categories of interest before training the system. In taxonomic identification, appropriate subdivisions might include sex (male or female), life stage, or color morph. For instance, assume that we are trying to separate two closely related species, $CDATA[$CDATA[$A$$ and $CDATA[$CDATA[$B$$, in a group with distinct sex dimorphism. Then it might be a good idea to train the system on separating males from females at the same time as it is trained to separate the species, as this will help the system in finding the sex-specific species differences between $CDATA[$CDATA[$A$$ and $CDATA[$CDATA[$B$$. Another idea is to combine images representing different viewing angles of the same specimen in the training set. For instance, the system might be trained on separating combined images showing both the dorsal and ventral side of the same specimen.

### Taxonomic Identification

Taxonomic identification tasks differ in several ways from more typical image classification problems. For instance, it is common that taxonomic image sets use a standard view, that specimens are oriented in the same way and occupy a large part of the frame, and that there are few disturbing foreign elements in the images. These are all features that should make it easier for machine learning algorithms to learn the classification task, since they reduce the variation within image categories. If images are more heterogeneous, like our data set D4, one expects more training data to be needed for the same level of identification accuracy. Interestingly, our results suggest that the extra difficulties introduced by more heterogeneous images are not as dramatic as one might think. Using roughly 400 images per category, we actually achieved better identification accuracies for the heterogeneous D4 than we did with the less challenging D1–D3 using 100–200 images per category.

Taxonomic identification tasks differ widely in the morphological variation one expects within categories, and the degree to which it is possible to cover that variation in the training set. It is perhaps not surprising that the task of identifying higher taxonomic groups is more challenging than the separation of species, even if the species are very similar to each other. Our data sets used for the identification of higher groups (D1–D2) are particularly challenging because a large fraction of the images (all in D1) belong to unique species. Thus, the model needs to be able to place specimens in the correct higher group even though it has not seen the species before, and possibly not the genus it belongs to either. This requires the model to achieve a good understanding of the diagnostic features of the higher taxonomic groups. In view of these challenges, the accuracies we report here on the higher-group identification tasks ($CDATA[$CDATA[$>$$92% for D1 and $CDATA[$CDATA[$>$$96% for D2) are quite impressive, especially given the small number of images per category in the training set. It requires a decent amount of training for a human to reach the same levels of identification accuracy. Given larger training sets, it seems likely that the automated systems would be able to compete successfully with experts on these tasks.

On the fine-grained tasks (D3–D4, D2B), our system clearly reaches or exceeds expert-level accuracies even with the small to modest training sets used here. On D3, the best taxonomists in the world could beat the automated system, but only if they had access to images of the ventral side of the specimens and locality of collecting. For a fair comparison, however, one would then also have to make ventral-view images available in the training set used for the automated system, which might tip the race again in the favor of machines.

On task D2B (separating two genera of Scarabaeidae), our automated system outperformed the expert we consulted, and on D4 it achieved significantly higher identification accuracies in separating the most challenging species pair than reported previously for 26 entomologists trained on the task (Larios et al. 2008) ($CDATA[$CDATA[$>$$98% vs. 78%). The human identification accuracies reported by Larios et al. may seem low, but previous studies have shown that humans are prone to make errors, regardless of whether they are experts or nonexperts (MacLeod et al. 2010; Culverhouse 2014; Austen et al. 2016 and references cited therein). The errors can be due to a variety of reasons: fatigue, boredom, monotony, imperfect short-term memory, etc. (Culverhouse 2007).

In many ways, our results demonstrate wide-ranging similarities between humans and machines in learning and performing taxonomic identification tasks. For instance, the amount of training is clearly important in improving machine performance (Fig. 7). Machine identification accuracies are significantly lower for groups that have not been seen much previously, and for entirely novel taxa (Fig. 8). Taxa that are unusually variable, such as the Tenebrionidae, are difficult to learn, while uniform groups that have striking diagnostic features, such as Pipunculidae, will be picked up quickly (Fig. 7).

It is also easy to recognize human-like patterns in the erroneous machine identifications. For instance, the D4 specimens that are misclassified by the automated system are unusually small, occluded, or imaged from a difficult angle (Fig. 11). These are specimens that humans would also have difficulties identifying correctly. Similarly, the errors in machine identification of *Oxythyrea* species are similar to the errors one might expect humans to make (Fig. 10).

Our results clearly demonstrate that modern machine learning algorithms are powerful enough to address even the most challenging taxonomic identification tasks. For instance, it appears worthwhile to test whether the method described here would also be able to identify cryptic species that are extremely challenging or impossible for human taxonomists to separate, such as bumblebee species of the *lucorum* complex (Scriven et al. 2015). In addressing such tasks, however, several problems might occur due to the difficulties of image annotation and the model’s “hunger for examples.” For instance, humans might not be able to provide accurate identifications without preparation of the genitalia or extraction of DNA for sequencing. Thus, accurate identification of the specimens may leave them in a condition making them unsuited for imaging, so unless they were imaged prior to identification, the specimens cannot be included in training sets.

An interesting aspect of taxonomic identification is that it falls into two distinct subdisciplines: field identification and identification of museum specimens. Even though the two are clearly separated, it is also obvious that knowledge developed in one domain will be useful in the other. Transferring knowledge between such related applications is known as *domain adaptation* in computer vision (Csurka 2017). This is a very active research topic, and although current techniques are less mature than the image classification algorithms used in this article, we might expect significant progress in the near future. It seems likely that citizen-science projects like iNaturalist and e-Bird will be able to generate large training sets of field photos of animals and plants in the near future, allowing the development of powerful automated systems for field identification. Efficient domain adaptation should make it possible to extract the machine knowledge about organism identification accumulated in such projects and use it in developing better systems for taxonomic identification of museum specimens. It may also be useful in many cases to transfer machine knowledge in the other direction.

## Conclusion

In conclusion, our results show that effective feature extraction from pretrained CNNs provides a convenient and efficient method for developing fully automated taxonomic identification systems. The general effectiveness of our approach on a wide range of challenging identification tasks suggests that the method can be applied with success even without task-specific optimizations. The method is robust to noise in the training set and yields good performance already with small numbers of images per category. Using the provided code, it should be easy to apply the method to new identification tasks with only some basic skills in python scripting.

With the advent of DL and efficient feature transfer from pretrained CNNs, the field of automated taxonomic identification has entered a completely new and exciting phase. Clearly, routine tasks of sorting and identifying biological specimens could be replaced already today in many situations by systems based on existing CNN technology. Actually, it may be time now to look beyond the obvious application of CNNs to routine identification, and start thinking about completely new ways in which CNNs could help boost systematics research.

One interesting application may be online discovery of new taxa as more and more of the world’s natural history collections get imaged and published on the web. Although detection of images representing potential new categories remains a challenging problem in computer vision, one can imagine a future in which a taxonomist would be alerted by an automated system when images of specimens that potentially represent new species of her group become available.

Using the high-level semantics of CNNs to generate natural-language descriptions of image features is another active research field in computer vision (Donahue et al. 2015; Karpathy and Fei-Fei 2015; Xu et al. 2015) that may have a dramatic impact on systematics. In theory, such systems could completely automate the generation of natural-language descriptions of new species.

The CNN-based automated identification systems clearly develop a powerful high-level understanding of the tasks with which they are challenged. If that understanding of taxonomic identification tasks could be communicated effectively to biologists, it could replace traditional identification keys. Perhaps the best way to do this would be through visualizations of morphospace that are optimized for identification. In other words, morphospace as it is organized by a CNN trained on a relevant identification task. It is not difficult to imagine ways in which such approaches might generate identification tools that would be highly efficient and very appealing to humans.

One could also imagine CNN-based systems for automating morphological phylogenetic analysis. Throughout the history of evolutionary biology, comparative anatomists and paleontologists have demonstrated that it is possible for the human brain to reconstruct phylogenies by intuitively evaluating a large body of morphological facts. In many cases, these morphology-based hypotheses have withstood the test of time even though they were challenged by many of the early molecular phylogenetic analyses. If it is possible for humans, it should also be possible for CNNs to learn how to infer phylogeny from morphology documented in images.

It is important for systematists to encourage and accelerate the collection and publishing of accurately labeled digital image sets, as the shortage of suitable training data is one of the major hurdles today for the development of computer-assisted taxonomic identification. The large museum digitization efforts that are under way in several countries will clearly benefit the development of automated identification systems, but more such projects are needed. The new identification systems, in turn, will help to significantly increase the impact of these imaging projects by using them to generate novel discoveries in taxonomy and systematics.

Clearly, morphology-based taxonomy and systematics are facing a period of transformative changes. By taking advantage of the new opportunities that are opening up thanks to developments in computer vision, systematists and taxonomists could accelerate the discovery of the planet’s biological diversity, free up more time to sophisticated research tasks, and facilitate the study of classical evolutionary problems, such as the evolution of morphological characters and the dating of divergence times using fossils.

## Supplementary Material

Data available from the Dryad Digital Repository: http://dx.doi.org/10.5061/dryad.20ch6p5 and https://github.com/valanm/off-the-shelf-insect-identification.

## Author Contributions

M.V., A.M., and F.R. conceived the idea; M.V., K.M., A.M., D.V., and F.R. designed the study; M.V. compiled and filtered data sets D1 and D2 and D.V. accumulated D3; M.V. wrote the scripts, performed analyses, interpreted results, and prepared the first draft. All authors revised and commented drafts at different stages and contributed to the final version of the manuscript.

## Funding

This work was supported by the European Union’s Horizon 2020 research and innovation program under the Marie Sklodowska-Curie [642241 to M.V.]; from Charles University [SVV 260 434/2018], from Ministry of Culture of the Czech Republic [DKRVO 2018/14, National Museum, 00023272], and from European Community Research Infrastructure Action under the Synthesys Project [FR-TAF-5869 (http://www.synthesys.info/)] to D.V.; and from the Swedish Research Council [2014-05901 to F.R.].
